# Evaluation of the Influence of Grain Sizes of Nanostructured WO_3_ Ceramics on the Resistance to Radiation-Induced Softening

**DOI:** 10.3390/ma16031028

**Published:** 2023-01-23

**Authors:** Dauren B. Kadyrzhanov, Artem L. Kozlovskiy, Maxim V. Zdorovets, Inesh E. Kenzhina, Dmitriy I. Shlimas

**Affiliations:** 1 Engineering Profile Laboratory, L.N. Gumilyov Eurasian National University, Nur-Sultan 010008, Kazakhstan; 2 Department of General Physics, Satbayev University, Almaty 050032, Kazakhstan; 3 Department of Intelligent Information Technologies, Ural Federal University, 620075 Yekaterinburg, Russia; 4 Advanced Electronics Development Laboratory, Kazakh-British Technical University, 59 Tole bi St., Almaty 050000, Kazakhstan

**Keywords:** WO_3_ ceramics, nanosized grains, hardening, radiation damage, strength, hardness

## Abstract

The main purpose of this study is to test a hypothesis about the effect of grain size on the resistance to destruction and changes in the strength and mechanical properties of oxide ceramics subjected to irradiation. WO_3_ powders were chosen as objects of study, which have a number of unique properties that meet the requirements for their use as a basis for inert matrices of dispersed nuclear fuel. The grain-size variation in WO_3_ ceramics was investigated by mechanochemical grinding of powders with different grinding speeds. Grinding conditions were experimentally selected to obtain powders with a high degree of size homogeneity, which were used for further research. During evaluation of the strength properties, it was found that a decrease in the grain size leads to an increase in the crack resistance, as well as the hardness of ceramics. The increase in strength properties can be explained by an increase in the dislocation density and the volume contribution of grain boundaries, which lead to hardening and an increase in resistance. During determination of the radiation damage resistance, it was found that a decrease in grain size to 50–70 nm leads to a decrease in the degree of radiation damage and the preservation of the resistance of irradiated ceramics to destruction and cracking.

## 1. Introduction

Alternative solutions in the field of the transition from and replacement of traditional nuclear fuel in order to reduce the production and accumulation of transuranium elements and nuclear waste is the use of dispersed nuclear fuel [1,2]. The concept of dispersed nuclear fuel is based on the replacement of traditional uranium fuel rods with assemblies consisting of ceramic materials based on oxides or nitrides that surround fissile nuclear materials [3,4]. The use of such assemblies makes it possible not only to switch to new fuel, but also to significantly increase the degree of its burnup, and as a result, increase the efficiency of reactors. Additionally, the use of dispersed nuclear fuel based on refractory oxide ceramics opens up prospects and opportunities for operating nuclear reactors at higher temperatures, including the creation of new types of high-temperature nuclear reactors [5,6,7].

In turn, the use of inert matrices based on oxide ceramics also makes it possible to reduce the amount of nuclear waste produced as a result of replacing uranium with plutonium, as well as to abandon a number of processes associated with the disposal of nuclear waste. In this regard, in the past few years, much attention has been paid to research related to the study of the prospects for the use of oxide refractory ceramics as a basis for inert matrices of dispersed nuclear fuel, as well as the development of methods for modifying these ceramics to increase resistance to radiation damage and their accumulation [8,9,10]. Thus, in some cases, to increase the resistance to radiation damage, it is proposed to use various variations of the compositions of ceramics obtained by doping, which makes it possible to increase their strength and mechanical properties [11,12,13,14]. At the same time, doping is a very expensive modification method and often does not meet expectations. Thus, for example, the use of doping oxide ceramics based on zirconium dioxide with such oxide compounds as magnesium or yttrium oxide, despite the increase in resistance to external influences, significantly complicates the technological process of creating ceramics, and can also lead to the formation of composition heterogeneities [15,16]. The use of aluminum oxide or yttrium oxide to stabilize nitride ceramics based on silicon nitride (Si_3_N_4_) or aluminum nitride (AlN) can lead to the fact that, with the accumulation of radiation damage, these stabilizing additives will serve as sinks for defects, which can lead to destabilization and the partial amorphization of the structure [17,18,19].

One of the simplest ways to increase resistance to mechanical damage and radiation degradation is to increase the strength properties of ceramics by hardening them, which can be achieved by varying the grain sizes that make up these ceramics [20,21]. This method is based on the hypothesis that with a decrease in the grain size, there is a natural increase in the dislocation density and grain boundaries, which, in turn, are restraining obstacles for propagation of cracks and chips during deformation and stress in the structure of the damaged layer [22,23,24]. At the same time, a change in grain size for refractory oxide ceramics can have a significant impact on their resistance to destruction, due to size and dislocation effects, which in turn will increase the service life of dispersed nuclear fuel.

The purpose of this study was to determine the effectiveness of grain-size reduction in WO_3_ ceramics for increases in the strength properties and stability of resistance to radiation damage and its accumulation. The choice of WO_3_ ceramics as objects of study is due to their physicochemical, heat-conducting, and strength properties, as well as the absence of polymorphic transformations in a wide temperature range [25,26]. The relevance of this study consists in the determination of the possibilities for a simple and affordable method for the modification of ceramics intended for materials in inert nuclear fuel matrices. As shown in a number of works [27,28,29], the transition to nanosized grains makes it possible to significantly increase the resistance to external influences and radiation damage due to changes in the grain boundaries and dislocation density. For example, it was shown in [29] that the transition to nanosized grains makes it possible to increase the resistance of ceramics to radiation damage, which, according to the authors, opens up prospects for the use of nanosized ceramics in the nuclear industry. It should also be noted that the resistance to radiation damage is influenced by many factors, including fracture toughness and resistance to cracking, which can be varied by changing the mechanical and strength properties of ceramics.

## 2. Experimental Section

Commercial WO_3_ microparticles with an average grain size of 1–1.5 µm purchased from Sigma Aldrich (Sigma Aldrich, St. Louis, MI, USA) were chosen as initial samples. By varying the grinding conditions, nanostructured ceramics with different grain sizes from 350 to 50 nm were obtained.

Nanostructured ceramics based on tungsten oxide (WO_3_) with different grain sizes were chosen as objects of study. The formation of nanostructured ceramics with specified grain-size parameters was carried out by mechanochemical grinding at different grinding speeds. A planetary mill PULVERISETTE 6 classic line (Fritsch, Berlin, Germany) was used for grinding. The grinding speed ranged from 250 to 600 rpm; grinding time was 5 h. The choice of grinding time was based on a priori experimental data, during which it was found that grinding the initial powdered ceramics for 5 h leads to the formation of nanosized particles with a homogeneity degree of more than 94%.

The grain sizes were determined using the optical laser diffraction technique implemented on an ANALYSETTE 22 NeXT Nano particle size analyzer (FRITSCH, Berlin, Germany). The size values of the obtained grains during grinding at different speeds were determined based on size charts, the analysis of which made it possible to determine the average grain size, homogeneity degree and standard deviation.

**Figure 1:**
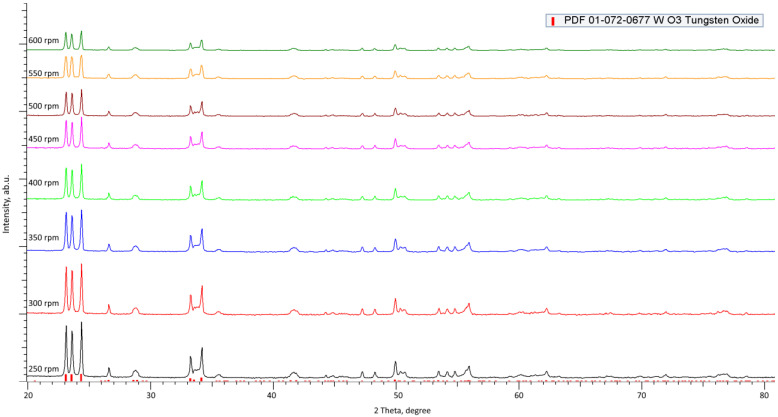
Results of X-ray diffraction of the studied ceramics.

Characterization of the stability of the phase composition of the selected ceramics depending on the grinding conditions was carried out using the X-ray phase analysis method implemented on a D8 Advance ECO powder diffractometer (Bruker, Berlin, Germany). X-ray diffraction patterns were taken in the range 2θ = 20–80°, with a step of 0.03°. Figure 1 shows X-ray diffraction patterns of the studied ceramic samples after grinding at different grinding speeds.

An analysis of the obtained diffraction patterns showed that the selected ceramics are characterized by a monoclinic WO_3_ phase (PDF-01-072-0677) with a high crystallinity degree (more than 90%).

As can be seen from the presented data, a change in the grinding rate does not lead to the formation or appearance of new diffraction reflections, which indicates that the mechanochemical grinding process itself does not lead to phase transformations or polymorphic transformations in ceramics. At the same time, the main changes with an increase in the grinding speed are associated with a decrease in the intensity of diffraction reflections and their slight shift. The change in the shape and intensity of reflections is due to the processes of change in the regions of coherent scattering (crystallite sizes), and the shift of reflections indicates the appearance of additional deformation distortions in the structure associated with a change in grain size.

To measure the strength properties, the resulting powders were pressed into tablets with a diameter of 10 mm and a thickness of 50 μm, which were subsequently used not only for mechanical testing, but also for irradiation. The determination of mechanical and strength properties was carried out using the following methods. The hardness of ceramic samples depending on the change in grain size was measured using the indentation method. For measurements, a LECO LM700 microhardness tester (LECO, Tokyo, Japan) was used. The Vickers pyramid was used as an indenter; the measurements were carried out with an indenter load of 100 N. All measurements were carried out serially (10–15 measurements) to establish the repeatability of hardness values.

Vickers hardness was calculated using Formula (1):

(1) $$HV=1.854\frac{P}{d^{2}},$$

where *P* is the applied pressure and d is the average length of the imprint diagonal. An estimate of the hardening value was calculated based on changes in hardness values depending on the grain size. All measurements were carried out on samples compressed into tablets.

The crack resistance of the samples was determined using the single compression method with fixed pressure value at which microcracks are formed. Fixation was carried out using a high-resolution extensometer.

The radiation-damage resistance was determined by irradiating the obtained samples with heavy Kr and Xe ions with energies of 150 and 230 MeV, respectively. Irradiation was carried out at the DC-60 heavy ion accelerator (Institute of Nuclear Physics, Astana, Kazakhstan). Irradiation of the samples was carried out in the form of tablets with a diameter of 10 mm and a thickness of 50 μm, which were obtained by pressing the obtained powders.

The choice of Kr and Xe heavy ions with the given energies is due to the possibility of modeling radiation damage processes in the near-surface layer of ceramics, comparable to fission fragments of nuclear fuel. The irradiation fluence was 10^15^ ion/cm^2^, the beam current was 10 nA, and the ion flux was 10^9^ ion/cm^2^ × s. The maximum path length of incident ions in ceramics was no more than 15 and 20 μm for Kr ions with an energy of 150 MeV and Xe ions with an energy of 230 MeV, respectively.

The choice of irradiation conditions and fluence was due to the possibility of modeling radiation damage in the material that occurs with a strong overlap of damaged areas along the trajectory of ions in the material.

## 3. Results and Discussion

### 3.1. Study of the Influence of Grinding Conditions on Grain Sizes

**Figure 2:**
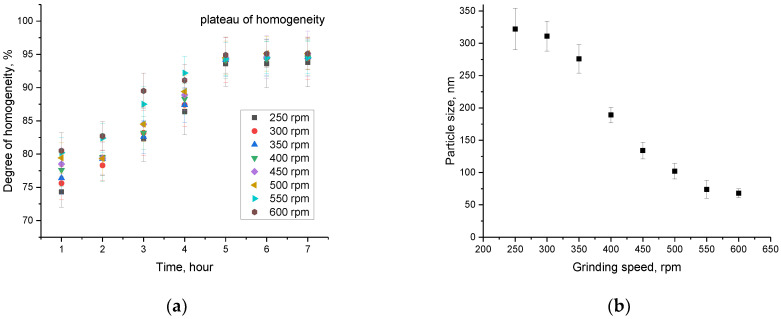
(**a**) Results of the homogeneity degree depending on the grinding conditions; (**b**) Results of particle size changes depending on the grinding speed.

Figure 2a shows the dependence of the change in the degree of homogeneity of particle sizes depending on the grinding time at different grinding speeds.

As can be seen from the data presented, with a grinding time of 1–3 h, the homogeneity degree of the obtained particle sizes, regardless of the grinding speed, is less than 80–85%, and the presence of large grains with a size of more than 500 nm was found in the resulting mixture itself. With an increase in grinding time above 4 h, the size homogeneity degree was 94–95%, and a further increase in grinding time does not lead to an increase in the homogeneity degree, on the basis of which the grinding time no more than 5 h was chosen.

Figure 2b shows the results of the change in particle size depending on the grinding speed. The data were obtained by analyzing the resulting powders using the optical laser diffraction method implemented on a particle analyzer. The average grain size, as well as the value of the standard deviation, was determined on the basis of the obtained size distribution diagrams and their subsequent analysis.

According to the data obtained, at a grinding speed of 250–300 rpm, the size of the resulting grains is at least 320–350 nm, while the observed mixture contains large grains with a size of more than 500 nm. Increasing the grinding speed above 300 rpm leads to a decrease in grain size to 150 nm, while the content of large fractions was less than 5%. In the case of a grinding speed above 500 rpm, the grain sizes in the mixture are less than 100 nm, which indicates nanostructured ceramics. Such a change in grain size because of a change in the grinding rate can be due to the processes of solid-phase synthesis. The grinding media acceleration leads to an increase in deformation in large grains, which is followed by their destruction into smaller ones, homogenization, and a decrease in the content of large inclusions. At the same time, an increase in the grinding speed from 550 to 600 rpm does not lead to large changes in the size of the resulting grains, which indicates the saturation limit of size reduction due to their crushing using mechanochemical mixing.

**Figure 3:**
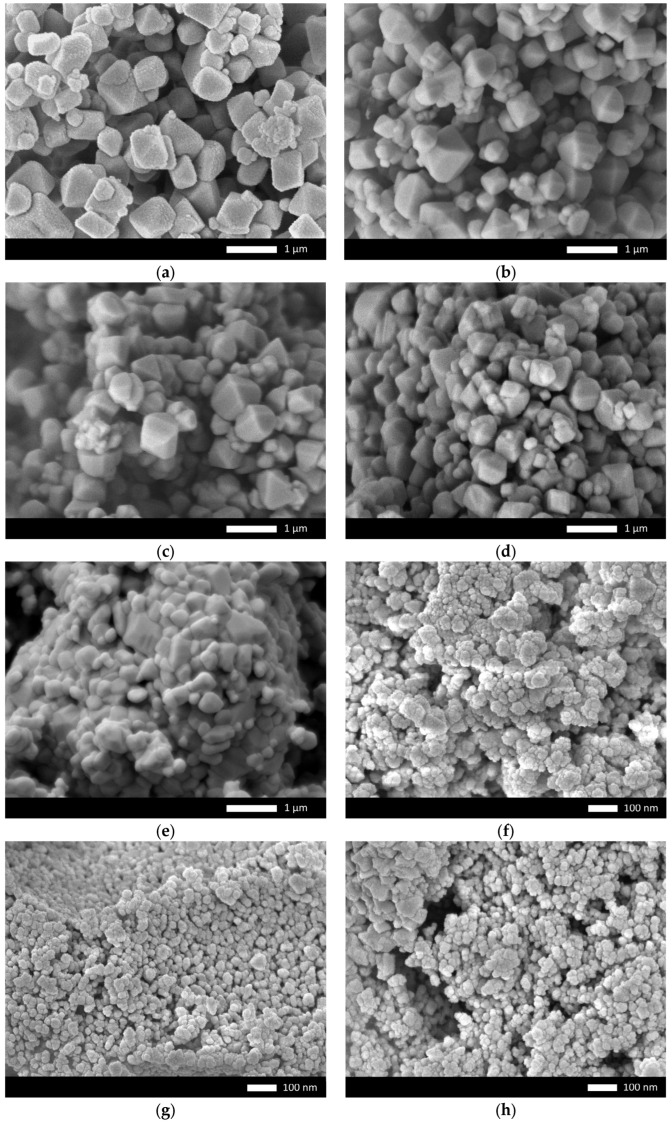
SEM images of the obtained ceramics depending on the grinding speed: (**a**) 250 rpm; (**b**) 300 rpm; (**c**) 350 rpm; (**d**) 400 rpm; (**e**) 450 rpm; (**f**) 500 rpm; (**g**) 550 rpm; (**h**) 600 rpm.

Figure 3 shows the SEM images of the obtained powders depending on the grinding speed, which are in good agreement in size with the data obtained using the laser optical diffraction method.

**Figure 4:**
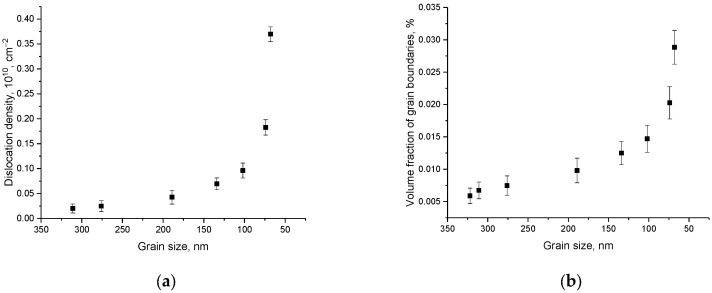
(**a**) Results of dislocation density change depending on grain size; (**b**) Evaluation results of the volume fraction contribution of grain boundaries in ceramics.

A change in the grain size, as a rule, is accompanied by the formation of additional boundaries and double and triple junctions, which can serve as sinks for defects and also affect the dislocation density (*δ*) [30,31]. At the same time, a hypothesis was previously put forward, according to which a decrease in grain size (*D*) with their transition to the nanoscale leads to the appearance of the effect of dislocation hardening. This hardening is due to a decrease in the grain size, and consequently, an increase in the dislocation density due to the inverse-square dependence of the dislocation density on the grain size (*δ = 1*/*D*^2^) [31]. At the same time, it is known that the dislocation density can also have both a hardening effect and lead to a deterioration in the resistance to destruction due to the formation of highly deformed regions and metastable inclusions in the structure. Figure 4a shows the results of the dislocation-density change depending on the grinding rate of ceramics, leading to a change in grain size. The dislocation density was calculated using the Williamson–Smallman relation, which can be written as *δ = 1*/*D*^2^ [31].

As can be seen from the data presented, the change in the dislocation density is most pronounced at high grinding rates, for which, according to the grain-size estimate, a decrease in grains and an increase in their homogeneity are observed. Moreover, these changes are most pronounced at a grinding speed of more than 500 rpm for which, according to the assessment of morphological features, the average grain size varies in the range of 70–100 nm. For given grain sizes, the dislocation density increases by a factor of 5–7 compared to coarse grains. Such a change in the dislocation density can lead to the creation of additional obstacles for microcracks and cleavages under external action on the ceramic, as well as increasing its resistance to destruction.

Another important factor that can have a significant impact on the hardening of materials, in addition to the dislocation density, is the factor associated with the grain boundaries and their volume contribution (*f_gb_*), leading to hardening due to the creation of additional obstacles in the material for point defects. To calculate the influence of the size factor associated with boundary effects, the following expression (2) can be used [32,33]:

(2) $$f_{gb}=\frac{3\delta \left(D^{2}-\delta^{2}\right)}{D^{3}},$$

where *D* is the grain size and *δ* is the grain boundary size (~0.1–0.5 nm). The evaluation results are presented in Figure 4b.

An analysis of the obtained data on the change in the volume fraction of grain boundaries, presented in Figure 4b, indicates the exponential nature of the change in the volume fraction with a change in the grinding speed, leading to a decrease in grain size. Moreover, low grinding speeds, leading to a slight change in grain size and a low homogeneity degree, do not lead to a significant increase in grain boundaries. In turn, a decrease in grain size leads to the formation of double-triple junctions, leading to an increase in boundary effects. At the same time, a decrease in the grain size below 100 nm leads to an increase in the volume fraction by almost an order of magnitude, which in turn can have a significant effect on the hardening efficiency.

**Figure 5:**
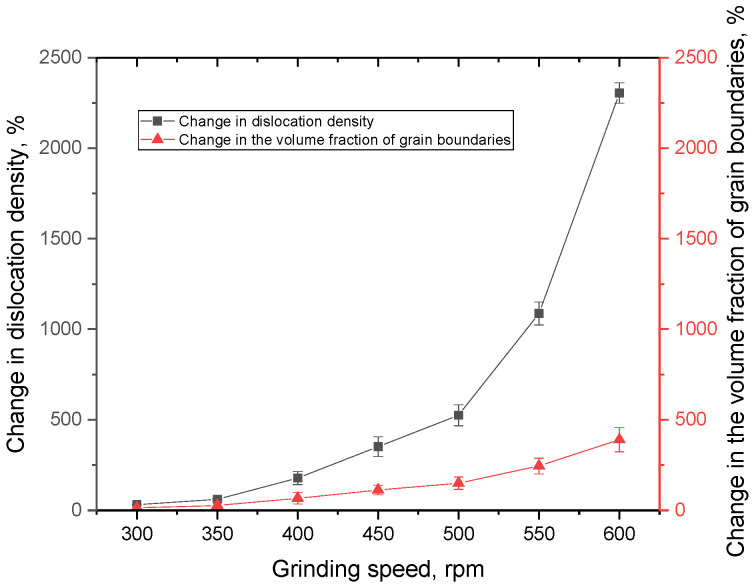
Evaluation results of the efficiency of the dislocation density and the volume fraction of grain boundaries change depending on grinding conditions.

Figure 5 presents comparative data on the efficiency (percentage ratio) of the change in dislocation density and the volume fraction of grain boundaries with a decrease in grain size, associated with a change in the grinding rate. These values characterize the dynamics of changes in the hardening factors in ceramics with a decrease in the size of the grains of which they are composed.

As can be seen from the data presented, the greatest change in size affects the change in dislocation density, which, when the grain size decreases to 100 nm, leads to a sharp increase in dislocation density (more than five times higher than the change in the volume fraction of grain boundaries). These differences can be explained by the fact that as the grain size decreases, the dislocation density, which has an inversely quadratic dependence on the size, sharply increases due to the small size, while the volume fraction has a more complex size dependence, which also includes the dimensions of the boundaries themselves.

### 3.2. Study of the Influence of Grain Sizes on the Hardening of Ceramics

One of the important factors that are key in this study is the determination of the efficiency of changing grain sizes in hardening ceramics to external mechanical influences under single compression, as well as determining the hardness of ceramics. As is known, a decrease in grain size can lead to the creation of additional barriers to the propagation of microcracks under external influences, which also increases the crack resistance. To measure the mechanical and strength properties, the obtained powders were pressed into tablets with a diameter of 8 mm and a thickness of 2 mm, which were subjected to further tests. To determine the changes in hardness, the indentation method was applied using a Vickers diamond pyramid. Based on changes in Vickers hardness values, the hardening coefficients of ceramics were calculated depending on the average grain size.

**Figure 6:**
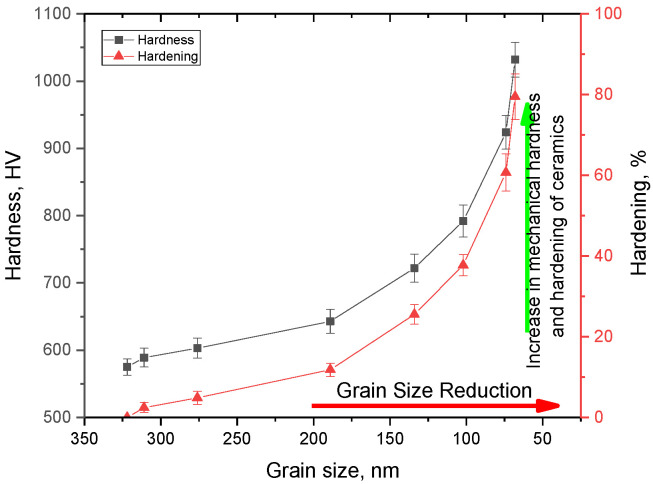
Results of changes in hardness and hardening of ceramics with a decrease in the grain size.

The data on the dependence of hardening on grain sizes presented in Figure 6 clearly demonstrate the effect of grain-size reduction on the increase in the strength properties of ceramics. As can be seen from the data presented, at grinding speeds of 250–400 rpm, which are characterized by fairly large grains, the change in hardness is no more than 3–5%, which indicates that hardening at large grain sizes is not strongly manifested. At the same time, a decrease in grain size to 150 nm or less leads to a sharp increase in hardness, the value of which increases by more than 30–50% compared with the same value for large ceramics. According to the data obtained, the main changes associated with an increase in the hardening coefficients calculated on the basis of changes in the hardness value occur when the grain size decreases below 200 nm. At the same time, the hardening value increases sharply in comparison with similar values for ceramics consisting of large grains (more than 250–300 nm). In this case, the maximum increase in hardness is observed for grains with a diameter of less than 100 nm.

**Figure 7:**
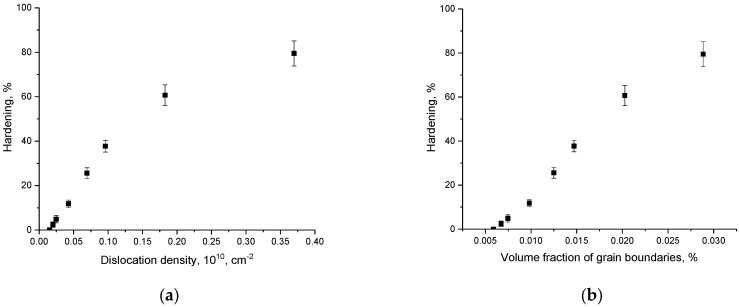
Results of a comparative analysis of the hardening coefficient depending on various hardening factors: (**a**) on the dislocation density (**b**) on the volume fraction of grain boundaries.

Figure 7 shows the dependencies of the hardening coefficients on the density of dislocations and the volume fraction of grain boundaries, which were calculated based on changes in grain sizes (see data in Figure 4a,b). As can be seen from the presented data, with small changes in grain sizes (at low grinding speeds), the greatest contribution to hardening is made by the effects of changes in dislocation density, while at small grain sizes, the main contribution to hardening is made by the effects associated with the volume fraction of grain boundaries.

**Figure 8:**
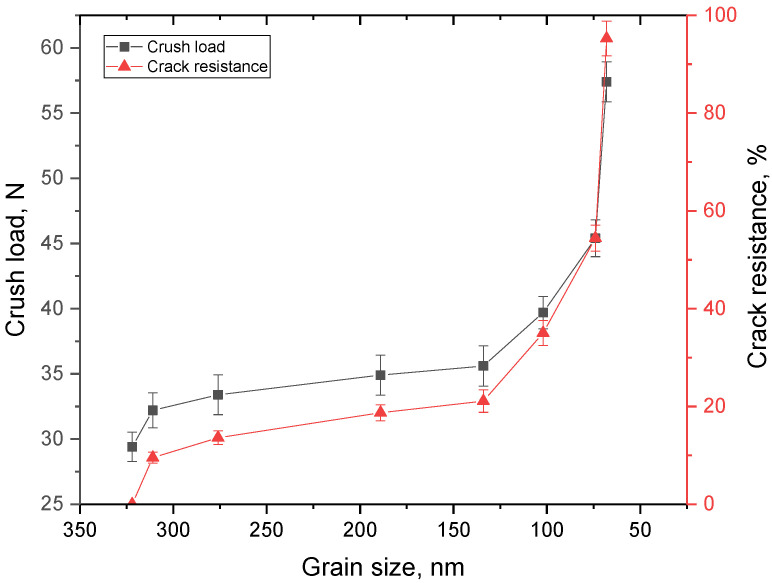
Results of changes in strength properties and crack resistance.

Figure 8 shows the dependences of the maximum pressure that the ceramics withstood under single compression, and the crack resistance of the ceramics on the grain size.

As can be seen from the data presented in Figure 8, in contrast to the change in hardness values, the most pronounced changes in crack resistance are observed with a decrease in size below 150 nm. At the same time, in the case of a decrease in size to 50–70 nm, it leads to an increase in crack resistance up to 80% in comparison with similar values for ceramics consisting of large grains. Such a difference in the nature of changes in the hardness of ceramics and their resistance to cracking can be explained by the fact that, during compression, cracks propagate along grain boundaries, which, in the case of small sizes, are quite numerous, and the rate of propagation of microcracks sharply decreases. When determining the hardness, resistance to pressure is exerted by dislocations, the density of which changes significantly even with a slight decrease in grain size (inverse quadratic dependence).

### 3.3. Determination of the Influence of Grain Sizes on Resistance to Radiation Damage

Thus, analyzing the data obtained, it can be concluded that a decrease in the grain size of ceramics has a positive effect on the hardening and resistance to cracking of ceramics. At the same time, an important factor in the case of the applicability of these ceramics as materials in inert matrices of dispersed nuclear fuel is the resistance to radiation damage processes and their accumulation, which can adversely affect the preservation of the strength properties of ceramics. As is known, the principle of dispersed fuel is based on the isolation of particles of fissile material (usually uranium or plutonium dioxide) from each other by an inert heat-conducting material—ceramics, which not only conduct heat, but also absorb most of the radiation damage caused by fission processes and nuclear reactions. At the same time, if the material of the inert matrix loses its strength properties, the processes of embrittlement and destruction of the material may occur, which in the most critical case can lead to the association of particles of the fissile material and an uncontrolled nuclear fission process. In this regard, a very important condition for inert matrices is to maintain the stability of its strength properties for a long time under prolonged exposure to radiation damage, including from fragments of the fission of nuclear fuel. Previously, in [27], it was shown that the greatest changes in radiation-damage resistance and a decrease in strength characteristics are observed at irradiation fluences of 10^14^–10^15^ ion/cm^2^ in the case of heavy Kr and Xe ions, comparable in energy and type to nuclear fuel fission fragments.

**Figure 9:**
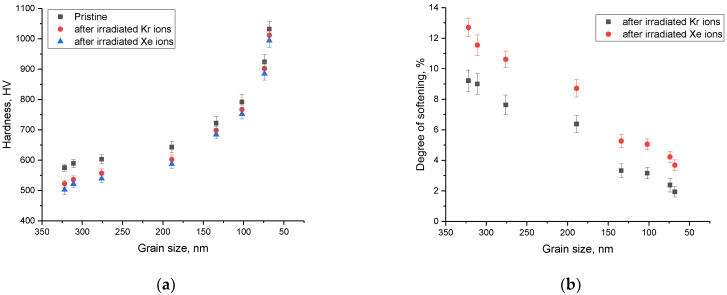
(**a**) Results of changes in the hardness of the near-surface layer before and after irradiation; (**b**) Results of changes in the near-surface layer softening value as a result of irradiation.

**Figure 10:**
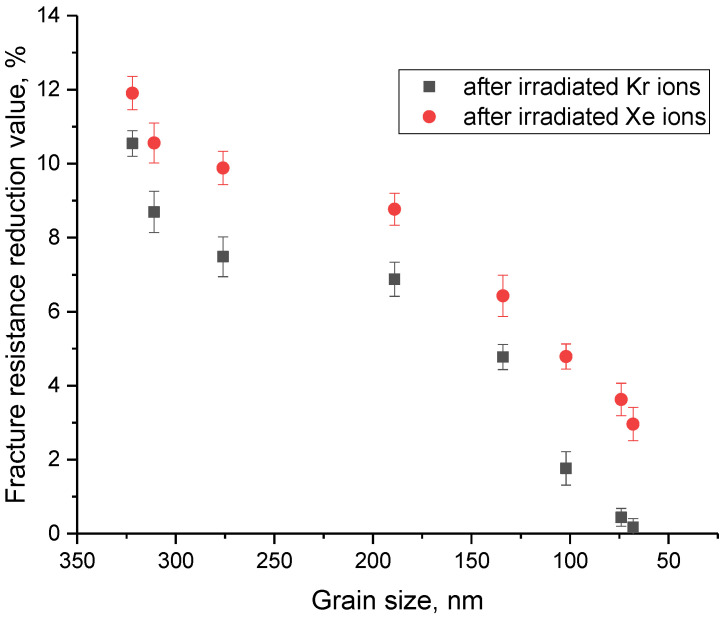
Results of changes in the crack resistance value of ceramics depending on the grain size for both types of irradiation.

To determine the efficiency of reducing the grain size of ceramics to increase resistance to radiation damage, the following experiment was carried out. Samples with different grains were irradiated with heavy Kr and Xe ions with energies of 150 and 230 MeV, respectively, with an irradiation fluence of 10^15^ ion/cm^2^. According to the results of several experimental works [27,34,35], this corresponds to the formation of highly defective regions in the structure of the damaged layer, which can lead to the destruction of the near-surface layer and its embrittlement. After irradiation, the microhardness values of the near-surface layer and the crack resistance were measured. Results of these experiments are shown in Figure 9. Figure 10 shows the results of assessing the change in the crack-resistance value for ceramics exposed to heavy ion irradiation.

The general view of the trends in the change in strength characteristics for samples irradiated with heavy Kr and Xe ions indicates that a decrease in grain size leads to an increase in resistance to radiation damage and the consequences caused by their accumulation. At the same time, the greatest effect of increasing the resistance to radiation-induced degradation of the near-surface layer of ceramics is manifested at small grain sizes, and also has a pronounced dependence on the type and energy of incident ions. This hardening can be explained by the following factors. During the interaction of incident heavy ions with a substance, the resulting point defects and vacancies have sufficient mobility and energy to migrate over the structure while forming agglomerates or clusters of defects. As a rule, such accumulation occurs near grain boundaries, which serve as so-called sinks of radiation defects. In turn, a decrease in grain size leads to the formation of a large number of grain boundaries, as well as an increase in their volume, which leads to the fact that the migration of point defects will be very difficult, and the probability of the formation of agglomerates or accumulations of defects is significantly reduced. At the same time, the energy of the incident ions also plays an important role in the processes of defect formation, since with an increase in the energy of the incident ions, the size of the damaged area along the trajectory of ions in the material increases, which, at high irradiation fluences, leads to an increase in the effect of overlapping radiation-damaged areas. An increase in the effect of overlapping of these areas leads to an increase in the destruction of the damaged near-surface layer, as well as its disordering. However, an increase in the dislocation density and volume fraction of grain boundaries leads to the formation of hardening effects, which significantly reduce the softening degree of the near-surface layer. In the case of a decrease in grain size to 50–70 nm, this leads to a decrease in the softening value by no more than 1.5–3%, depending on the type of ions. At the same time, the change in crack resistance for ceramic samples consisting of grains of 50–70 nm after irradiation with heavy ions was less than 1%, which indicates a high resistance to the formation of microcracks in irradiated samples.

## 4. Conclusions

Thus, by analyzing the data obtained on the values of the hardening and crack resistance of ceramics, it can be concluded that a decrease in grain size in ceramics below 100 nm is a very good deterrent to softening and destruction upon irradiation. Such an increase in resistance to radiation and mechanical damage for nanostructured ceramics can be explained by hardening effects associated with changes in the dislocation density and volume fraction of grain boundaries. A decrease in the grain size leads to the hardening of ceramics, as well as an increase in the resistance to destruction of the near-surface layer during the accumulation of radiation damage, which in turn increases the prospects for using these types of ceramics as the basis for inert matrix materials.

During the studies to determine the effect of grain sizes on resistance to radiation damage and the preservation of strength properties, the following was established. A decrease in grain size leads to an increase in resistance to mechanical damage in irradiated samples, and in the case of small sizes, the hardness values remain practically unchanged after irradiation. Thus, we can conclude that the transition to nanosized grains makes it possible to increase not only the strength properties of ceramics, but also increase the resistance to radiation damage when irradiated with heavy ions, comparable to nuclear fuel fission fragments.

The data obtained will be further used to conduct experiments to determine the efficiency of using these ceramics as dispersed nuclear fuel, as well as to conduct experiments to assess the swelling resistance of these ceramics. The established dependences of grain sizes on increasing radiation-damage resistance, as well as to mechanical influences, open up great prospects for development in this direction.

## Data Availability

Not applicable.
